# Intravitreal conbercept for branch retinal vein occlusion induced macular edema: one initial injection versus three monthly injections

**DOI:** 10.1186/s12886-020-01494-x

**Published:** 2020-06-11

**Authors:** X. Chen, T. M. Hu, J. Zuo, H. Wu, Z. H. Liu, Y. X. Zhan, Y. Xia, J. Wang, W. Wei

**Affiliations:** Jiangsu Province Hospital of Chinese Medicine, Affiliated Hospital of Nanjing University of Chinese Medicine, Nanjing, 210000 China

**Keywords:** Branch retinal vein occlusion, Macular edema, Conbercept, PRN

## Abstract

**Background:**

To compare the efficacy of one initial intravitreal injection of conbercept (IVC) versus three monthly IVCs in patients with macular edema (ME) after branch retinal vein occlusion (BRVO). Both options were followed by a pro re nata (PRN) retreatment regimen.

**Methods:**

This study retrospectively investigated and followed 60 patients with acute ME secondary to BRVO for over a year. 30 subjects received one initial injection (1 + PRN group); while, 30 received three monthly injections (3 + PRN group). The functional and anatomic outcomes were assessed during each follow-up.

**Results:**

The general characteristics of the 60 subjects were as follows: mean [SD] age, 57.43 [13.06] years; 33 [55%] female; 36 [60%] non-ischemic form. Both groups showed a stable gain in visual acuity (VA) with similar logMAR (mean ± SD) (1 + PRN group 0.308 ± 0.399, 3 + PRN group 0.34 ± 0.352) during the first 12 months. Additionally, both groups exhibited a significant reduction in central foveal thickness (CFT) with no statistically significant difference between them (1 + PRN group 222.1 μm ± 197.1 *μm*, 3 + PRN group 228.4 *μm* ± 200.2 *μm*). Both treatment groups had similar improvements in logMAR and anatomic outcomes over time. The stratified analysis showed that patients with the non-ischemic form and those with the ischemic form had similar improvements in VA (0.346 ± 0.366 VS 0.29 ± 0.39, *P* = 0.575) during the 12 months follow-ups. The number of injections was lower in the 1 + PRN group (4.0 ± 1.6) than in the 3 + PRN group (4.7 ± 1.3) (*P* = 0.068). No adverse effects or unexpected safety issues were reported in either group.

**Conclusions:**

Conbercept yielded significant improvements in VA and CFT among patients with BRVO induced ME, independent of their retinal ischemia status. The results showed that the 3 + PRN regimen do not lead to better functional outcomes or lower treatment needs in clinical practice as compared to the 1 + PRN regimen.

## Background

Retinal vein occlusion (RVO) is the second most common retinovascular disease after diabetic retinopathy, resulting in severe visual dysfunction among several patients worldwide [1]. A previously conducted pooled analysis showed that branch retinal vein occlusion (BRVO) (0.44%) had a higher prevalence than central retinal vein occlusion (CRVO) (0.08%) [2]. Clinically, observations of BRVO by fundus photography and fluorescein angiography are often indicative of retinovascular hemorrhage, dilated and tortuous retinal vessels, cotton-wool spots, and retinal non-perfusion (NP).

Vascular endothelial growth factor (VEGF), through its complex interactions with vascular endothelial cell proliferation and vascular permeability, plays a key role in the pathogenesis of BRVO induced macular edema (ME) [3] and neovascularization [4, 5]. It has been well established that retinal nonperfusion and hemorrhages due to vascular occlusion can lead to an increase in VEGF production, hence exacerbating ME and ischemia [6]. In recent years, the use of anti-VEGF inhibitors has dramatically increased, making them the standard of care for BRVO-ME in many countries [7]. Studies have shown that intravitreal injections of anti-VEGF inhibitors are more effective in resolving ME and improving the best-corrected visual acuity (BCVA), including bevacizumab [8], ranibizumab [7], or aflibercept [9]. Conbercept (KH902; Chengdu Kanghong Biotech Co., Ltd., Sichuan, China) has been approved by the Chinese Food and Drug Administration for the treatment of RVO induced ME. It is a fusion protein that contains the extracellular domain 2 of VEGF receptor 1 and extracellular domains 3 and 4 of VEGF receptor 2 combined with the Fc portion of the human immunoglobulin G1. Previous studies have also shown that a regular pro re nata (PRN) use of VEGF blockade in RVO seems to have a significant and lasting effect on VA gain [10]. YUKO MIWA et al. [11] showed that there are no significant differences in functional outcomes between BRVO induced ME patients treated with a single monthly PRN injection of ranibizumab and those with three monthly PRN injections. The efficacy and safety of 3 monthly PRN injections of conbercept for the treatment of ME after RVO have been previously established by FALCON et al. [12]. Therefore, we evaluated the benefits and compared the visual and anatomical outcomes of a single monthly injection versus three monthly injections of conbercept in the treatment of ME after BRVO.

## Methods

### Participants

This retrospective study analyzed data from 60 patients with a confirmed diagnosis of macular edema after BRVO and a history of intravitreal injections of conbercept. The data in this study was collected from August 2017 to December 2019. All data originated from the Department of Ophthalmology, Jiangsu Provincial Hospital of Chinese Medicine (Nanjing, China). The purpose of the treatment and potential adverse effects were thoroughly explained, and all subjects signed informed consent before treatment initiation.

The eligibility criteria included the followings: patients should be at least 18 years of age; have decreased visual acuity; should have BRVO with retinal edema, and a central foveal thickness CFT > 250 μm as assessed by optical coherence tomography (OCT). The exclusion criteria were: patients with their last intravitreal anti-VEGF or steroids treatment being within less than 6 months; patients with a history of ocular surgery; patients with an ocular disease such as diabetic retinopathy, senile cataract, and age-related macular degeneration, that significantly affected the BCVA; patients with a history of interventions or neovascularization (NV) before the study period; patients with advanced glaucoma, intraocular pressure (IOP) > 22 mmHg.

### Treatment

The study sample collected devided into either the 1 + PRN group or the 3 + PRN group. 30 eyes received a single monthly intravitreal injection of conbercept, and 30 eyes received three monthly intravitreal injections of conbercept. An initial intravitreal conbercept injection was administered at day one, followed by a continuous monthly treatment until stabilization of BCVA. The retreatment criteria, according to the RPN scheme, were: vision loss of ≥10 ETDRS letters compared with the previous month’s BCVA; increase of ancentral subfield thickness (CST) ≥ 50 μm; CST > 340 μm; presence of intraretinal fluid, intraretinal cysts, or subretinal fluid ME [13].

### Assessments

The Early Treatment Diabetic Retinopathy Study (ETDRS) was used to evaluate temporal changes in best-corrected VA between the two groups. The secondary outcome was defined as pre and post-treatment mean changes in central retinal thickness (HD-OCT (Zeiss, Germany)) and is reflective of treatment response. The CFT was assessed by HD-OCT measurements between the vitreoretinal interface and the anterior boundary of the retinal pigment epithelium. Additional outcomes were defined as follows: the investigation and evaluation by fluorescein fundus angiography (FFA, Heidelberg, Germany) of ischemic or non-ischemic BRVO and retinal circulation; the analysis and quantification of the number of injections during the 12 months follow-up. Safety assessments, including ocular, systemic adverse events (AEs), serious AEs (SAEs), and their correlation with the treatment, as well as injection procedures, were carried out at each follow-up.

### Statistical analysis

All statistical analyses were carried out using SPSS 20.0 (SPSS, Inc., Chicago, IL, USA). Quantitative data were presented as mean ± SD. VA measurements were converted to a logarithm of the minimum angle of resolution (logMAR) for statistical analysis. A paired t-test was used to analyze changes in VA as well as CFT within each group. Meanwhile, an unpaired t-test was used to compare the two parametric data between treatment groups. Differences in distributions were analyzed through chi-square tests. A *P* value < 0.05 was considered statistically significant.

## Results

### Study population and baseline characteristics

Among the 60 participants in this study, 30 (50%) received a single initial IVC, and 30 (50%) received three monthly IVC. The baseline characteristics of each study group are summarized in Table 1. There were similarities in patient demographics and baseline ocular characteristics between the two groups. 36 eyes were classified as non-ischemic, and 24 eyes were classified as ischemic after FFA. All patients in this study are Asian.

**Table 1:** Baseline Characteristics of the Study Population

	*Total*	*1 + PRN Group*	*3 + PRN Group*	*P*
*Baseline*				
Number (patients/eyes)	60/60	30/30	30/30	NA
Gender(Male/ Female)	27/33	15/15	12/18	0.436
Age, years	57.43 ± 13.06	57.33 ± 14.47	57.53 ± 11.73	0.953
Diagnosis (non-ischemic/ ischemic)	36/24	16/14	20/10	0.292
BCVA (LogMAR)	0.758 ± 0.413	0.77 ± 0.371	0.746 ± 0.466	0.823
CFT, μm	520.4 ± 186.5	522.4 ± 191.5	518.4 ± 184.5	0.935
*Final*				
BCVA (LogMAR)	0.434 ± 0.283	0.462 ± 0.261	0.405 ± 0.306	0.445
CFT, μm	295.1 ± 71.2	300.2 ± 89.3	290 ± 47.7	0.580
Number of injections during 12 mo	4.4 ± 1.5	4.0 ± 1.6	4.7 ± 1.3	0.068

*PRN* pro re nata, *BCVA* best-corrected visual acuity, *LogMAR* the logarithm of the minimum angle of resolution, *CFT* Central foveal thickness

Continuous variables were presented as mean and SD

*P*-value < 0.05 was considered statistically significant

### Change in best-corrected visual acuity and central foveal thickness

**Fig. 1:**
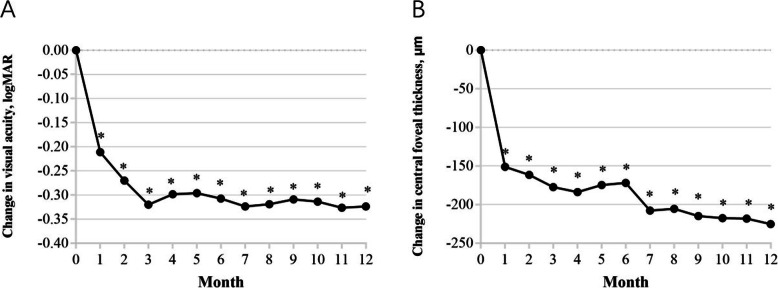
Changes in mean visual acuity (logMAR) and CFT in all treated eyes from baseline to month 12. The results indicated a significant improvement in mean VA (0.324 ± 0.373) from the baseline to month 12. Similarly, the results indicated a significant decrease in CFT (225.3 μm ± 197 μm) from the baseline to month 12. ^*^p = 0.000 compared with baseline values

**Fig. 2:**
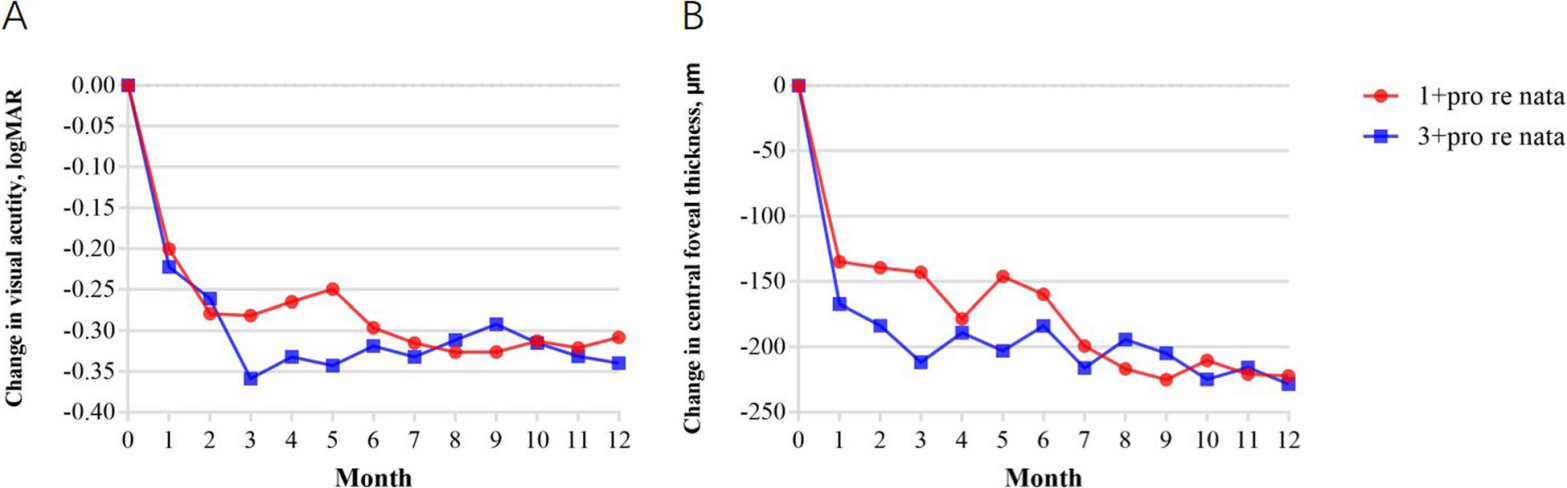
Changes in mean visual acuity (logMAR) and CFT from baseline to month 12. The results showed subsequent improvement in visual acuity with a rapid reduction in CFT after conbercept injections. Mean VA were 0.308 ± 0.399 in the 1 + PRN group and 0.34 ± 0.352 in the 3 + PRN group at baseline. The results also indicated a significant decrease in CFT (222.1 μm ± 197.1 *μm* for the 1 + PRN group and 228.4 μm ± 200.2 μm for the 3 + PRN group) after initiation of the treatment. The differences in CRT and VA between the two groups were not statistically significant (*P* > 0.05)

Both groups showed significant improvement in mean BCVA and reduction in mean CFT one month after the initiation of the treatment. Figure 1 shows significant improvements in overall mean BCVA (0.324 ± 0.373) from the baseline values to the 12 months follow-up in all treated eyes (*P* < 0.001). The 12 months follow-up indicated a significant reduction in overall mean CFT (225.3 μm ± 197 μm) of all treated eyes (*P* < 0.001). Figure 2 indicates an increase in means BCVA (0.308 ± 0.399 logMAR and 0.34 ± 0.352 logMAR) at 12 months in the 1 + PRN and the 3 + PRN groups, respectively. No statistically significant difference was seen between the two groups (*p* = 0.741). Both groups showed a significant decrease in means CFT (222.1 μm ± 197.1 μm and 228.4 μm ± 200.2 μm) at the 12 months follow-up, with no statistically significant difference between them (*p* = 0.902). A secondary analysis indicated that CFT tended to be smaller and VA to be better in the 3 + PRN group compared to the 1 + PRN group in the 12 months follow-up. Additionally, the analysis indicated that the choice of the treatment regimen was no association with the final BCVA and final CFT (*P* > 0.05).

### Stratified analysis of best-corrected visual acuity and central Foveal thickness

**Fig. 3:**
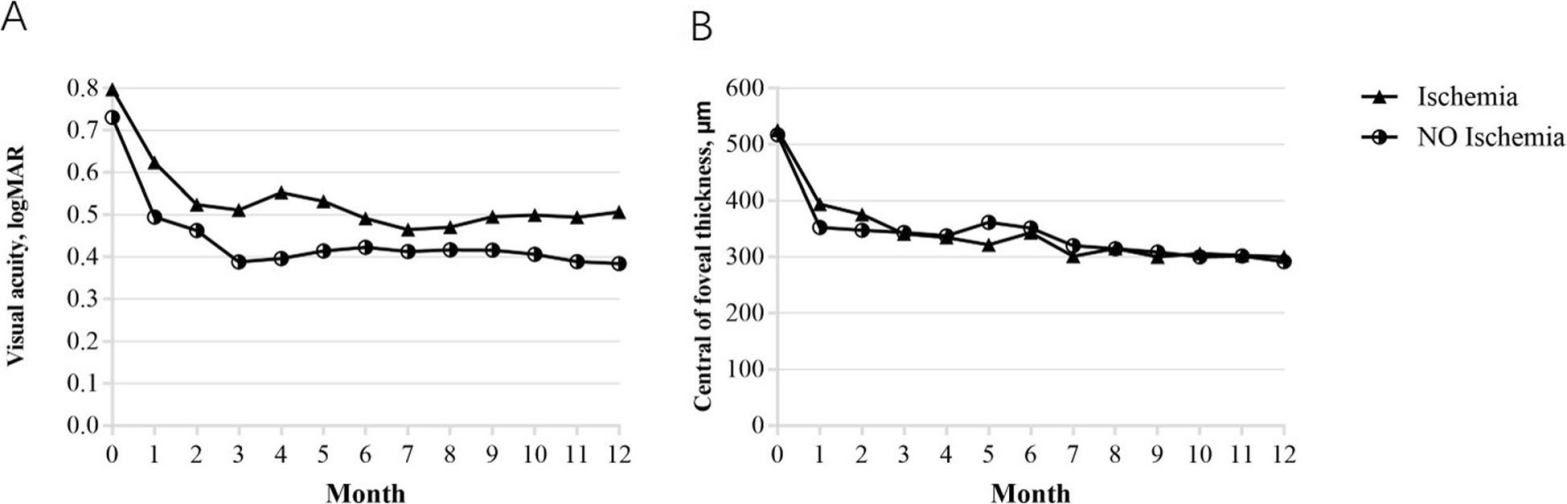
Changes in mean visual acuity (logMAR) and CFT in the ischemic and non-ischemic subgroups from baseline to month 12. Mean VA gained from 0.731 ± 0.347 at baseline to 0.385 ± 0.219 at month 12 in the non-ischemic subgroup, and improved from 0.798 ± 0.502 at baseline to 0.507 ± 0.352 at month 12 in the ischemic subgroup (*p* < 0.001 and *P* = 0.001, respectively). Similarly, mean CFT reduced from 517.3 μm ± 205.2 *μm* at baseline to 291.8 *μm* ± 75.9 *μm* at month 12 in the non-ischemic subgroup, and 525.1 *μm* ± 158.5 *μm* at baseline to 300.1 *μm* ± 64.7 *μm* at month 12 in the ischemic subgroup (*p* < 0.001)

We stratified patients according to baseline FFA examinations. Patients with a peripheral capillary nonperfusion greater than five disc areas were categorized as ischemic BRVO (*n* = 24 eyes), and the remaining were classified as non-ischemic BRVO (*n* = 36 eyes). Figure 3 shows the mean CFT and the logMAR VA in each subgroup. The findings indicated significant improvements in VA (0.346 ± 0.366 logMAR in the non-ischemic and 0.29 ± 0.39 logMAR in the ischemic subgroups) at the 12 months follow-up. However, no statistically significant difference was observed between the two subgroups (*P* = 0.575). Even so, the non-ischemic subgroup exhibited better mean VA than the ischemic subgroup during the entire observation period. Similarly, a significant decrease in CFT (225.5 μm ± 219.8 μm in the non-ischemic subgroup and 225 μm ± 161.3 μm in the ischemic subgroup) was seen at the 12 months follow-up. Nevertheless, there was no statistically significant difference between the two subgroups (*P* = 0.993).

### Number of pro re nata Conbercept injections

**Fig. 4:**
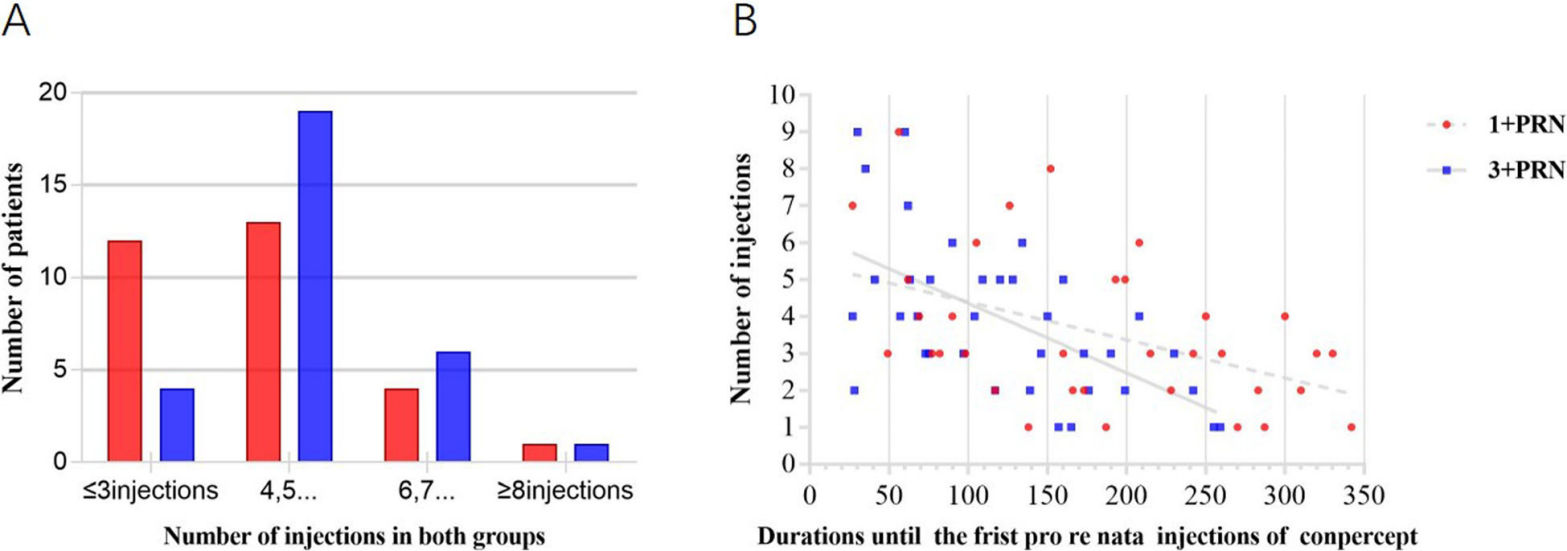
B. Scatter plots showing the association between the number of PRN conbercept injections and the duration until the first PRN injection. The total number of PRN injections was significantly associated with shorter durations until initial PRN injection in both groups (*R* = -0.459, *P* = 0.006, *R* = -0.6, *P* = 0.000, respectively)

Over the 12 postoperative months, the mean ± SD number of conbercept injections in patients receiving the 1 + PRN and the 3 + PRN (range 2–9) were 4.0 ± 1.6 and 4.7 ± 1.3, respectively. There was no significant difference between the two groups (*P* = 0.068, Table 1). The total number of PRN injections was significantly associated with a shorter duration until the first PRN injection in both groups (*R* = -0.459, *P* = 0.006, *R* = -0.6, *P* = 0.000, respectively, Fig. 4b).

### Safety assessments

Adverse events of interest were rare during the entire observation period, and all patients in the study did not show significant side effects.

## Discussion

Although the traditional PRN treatment regimens have demonstrated their usefulness in the treatment of BRVO, the debate of whether their efficacy outways the resultant patient burden on the health care system remains. In recent years, anti-VEGF intravitreal therapy has been extensively used for the treatment of ME associated with BRVO. This study was conducted to assess the effects of monthly intraocular injections of conbercept through a span of 12 months. According to our results, even though the overall mean number of injections was 4.4 ± 1.5, the use of conbercept with PRN regimens yielded favorable outcomes in visual acuity and steady reductions in ME.

The overall sample is comprised of 60 eyes, with 30 eyes allocated to the 1 + PRN group and 30 eyes to the 3 + PRN group. The results showed that VA and CFT at baseline were not significantly different between the two treatment groups. Further analysis indicated that the 1 + PRN group showed significant improvement in mean logMAR VA (from 0.77 ± 0.371 at baseline to 0.462 ± 0.261 at month 12 (*p* < 0.001)) at 12 months follow-up. Similarly, the 3 + PRN group also exhibited improvements in mean logMAR VA (from 0.746 ± 0.466 at baseline to 0.405 ± 0.306 at month 12 (*p* < 0.001)) during the last follow-up. Additionally, both groups showed significant decrease in mean CFT (from 522.4 μm ± 191.5 μm at baseline to 300.2 μm ± 89.3 μm at month 12 (p < 0.001) for the 1 + PRN group and from 518.4 μm ± 184.5 μm at baseline to 290 μm ± 47.7 μm at month 12 (*p* < 0.001) for the 3 + PRN group, respectively) during the last follow-up. There was no significant difference in the final mean VA or final CFT between the two regimens. The total number of injections per eye in the 1 + PRN group (4.0 ± 1.6) was slightly lower than that in the 3 + PRN group (4.7 ± 1.3) (*P* = 0.068). A reason for this may be that conbercept displayed long half-life and strong bioavailability [14]. 1 + PRN regimen could support a prolonged dosing interval than 3 + PRN regimen in disease stability criteria. However, patients tended to have greater CFT and worse VA in the 1 + PRN group as compared to the 3 + PRN group during the observation period (although the changes of VA and CFT were not statistically significant between the two groups). Moreover, lower fluctuation of mean logMAR VA and CFT were observed in patients belonging to the 3 + PRN group at the 12 months follow-up. ZUHUA SUN et al. [12] previously reported that the differences in mean VA values between BRVO and CRVO groups were relatively small (17.83 ± 10.89 letters VS 14.23 ± 11.74), indicating that IVC is generally safe and effective. Thus, taking into account our findings, it is clear that the reductions in mean CFT and gains in mean VA in the 1 + PRN regimen are almost similar to those of the 3 + PRN regimen. A recent study [15] with similar study designs and using PRN treatment protocols also had similar outcomes. Both regimens showed similar efficacy.

Our study not only included patients with PRN treatment regimens but also those with non-ischemic or ischemic BRVO induced ME. We observed higher injection numbers on average in eyes belonging to the ischemic subgroup (4.6 ± 1.7) as compared to those in the non-ischemic subgroup (4.2 ± 1.3, *P* = 0.308). The baseline data showed that compared to the 36 non-ischemic eyes, the 24 eyes in the ischemic subgroup had slightly worse CFT and VA values. However, the difference was not statistically significant. This may be partially due to the correlation between ischemia and greater CFT, as well as poorer VA [16]. Stratified analysis of the non-ischemic subgroup showed a rapid reduction in ME and significant improvement of BCVA at month 12 (from 0.731 ± 0.347 at baseline to 0.385 ± 0.219, *p* < 0.001). In contrast, the analysis of the ischemic subgroup indicated a reduction in ME with slow improvement in VA at month 12 (from 0.798 ± 0.502 at baseline to 0.507 ± 0.352, *P* = 0.001). Additionally, VA tended to be lower in the ischemic subgroup as compared to the non-ischemic one. Consequently, the findings suggested that these factors are associated with the visual functional outcome.

It is worth noting that the novelty of this study is that it provides crucial information on anti-VEGF therapy and shows that it can achieve a promising outcome and significantly improve VA and CFT regardless of the nature of the ME (non-ischemic or ischemic). In this study, 10 of the ischemic subgroup patients received conbercept +laser treatments. Interestingly, The BRIGHTER study has previously shown that the addition of laser does not lead to better functional outcomes or lower treatment needs [17]. In their study, Ramin Tadayoni et al. compared changes in BCVA outcome and ranibizumab injections between the ranibizumab + laser and the ranibizumab monotherapy groups. They concluded that there were no significant differences in VA and numbers of ranibizumab injections between groups (11.3 vs. 11.4). Another recent clinical trial [18] demonstrated that the trend of improvement in VA and reduction of CMT observed during conbercept usage for the treatment of ME secondary to BRVO were similar to those obtained with ranibizumab.

In summary, our study reported findings on the efficacy of anti-VEGF treatment and provided additional data on PRN protocols in patients with BRVO-ME. It showed that there were no significant differences in outcomes between the 1 + PRN and the 3 + PRN groups. Additionally, visual stabilization was observed after a prolonged treatment period and regular anti-VEGF dosing, hinting that the adoption of such protocol might help reduce the treatment burden. One of the major limitations of this study is its small number of participants and its relatively short follow-up period. PRN regimens are likely to be beneficial for patients with such conditions. However, future research should focus on larger controlled studies and long-term dosing strategies to confirm our data.

## Conclusions

This observational study confirms that Conbercept resulted in clinically and statistically significant visual and anatomic benefits for patients with ME secondary to BRVO. Three monthly injections might be more appropriate with patients even though no significant differences between the 1 + PRN and the 3 + PRN groups.

## Data Availability

The datasets generated and/or analysed during the current study are not publicly available due to the prevision of further publications coming soon, but are available from the corresponding author on reasonable request.

## Abbreviations

BRVO: Branch retinal vein occlusion
IVC: Intravitreal injection of conbercept
ME: Macular edema
PRN: Pro re nata
BCVA: Best-corrected visual acuity
logMAR: Logarithm of minimum angle of resolution
CFT: Central foveal thickness
OCT: Optical coherence tomography
FFA: Fluorescein fundus angiography
ETDRS: The Early Treatment Diabetic Retinopathy Study
SD: Standard deviation
